# Effects of alpha-calcitonin gene-related peptide on osteoprotegerin and receptor activator of nuclear factor-κB ligand expression in MG-63 osteoblast-like cells exposed to polyethylene particles

**DOI:** 10.1186/1749-799X-5-83

**Published:** 2010-11-04

**Authors:** Jie Xu, Max D Kauther, Julia Hartl, Christian Wedemeyer

**Affiliations:** 1 Department of Orthopaedics, University of Duisburg-Essen, Pattbergstrasse 1-3, 45239 Essen, Germany; 2 Department of Orthopaedics, The second affiliated hospital of Sun Yat-sen University Guangzhou, PR China; 3 Department of Trauma Surgery, University of Duisburg-Essen, Hufelandstraße 55, 45147 Essen, Germany

## Abstract

**Background:**

Recent studies demonstrated an impact of the nervous system on particle-induced osteolysis, the major cause of aseptic loosening of joint replacements.

**Methods:**

In this study of MG-63 osteoblast-like cells we analyzed the influence of ultra-high molecular weight polyethylene (UHMWPE) particles and the neurotransmitter alpha-calcitonin gene-related peptide (CGRP) on the osteoprotegerin/receptor activator of nuclear factor-κB ligand/receptor activator of nuclear factorκB (OPG/RANKL/RANK) system. MG-63 cells were stimulated by different UHMWPE particle concentrations (1:100, 1:500) and different doses of alpha-CGRP (10^-7 ^M, 10^-9 ^M, 10^-11 ^M). RANKL and OPG mRNA expression and protein levels were measured by RT-PCR and Western blot.

**Results:**

Increasing particle concentrations caused an up-regulation of RANKL after 72 hours. Alpha-CGRP showed a dose-independent depressive effect on particle-induced expression of RANKL mRNA in both cell-particle ratios. RANKL gene transcripts were significantly (P < 0.05) decreased by alpha-CGRP treatment after 48 and 72 hours. OPG mRNA was significantly down-regulated in a cell-particle ratio of 1:500 after 72 hours. Alpha-CGRP concentrations of 10^-7 ^M lead to an up-regulation of OPG protein.

**Conclusion:**

In conclusion, a possible osteoprotective influence of the neurotransmitter alpha-CGRP on particle stimulated osteoblast-like cells could be shown. Alpha-CGRP might be important for bone metabolism under conditions of particle-induced osteolysis.

## Background

Mechanical wear in the joint of a total hip replacement is responsible for a severe inflammatory reaction due to the release of cytokines and other soluble mediators that favor osteoclast generation, bone resorption and, in turn, prosthetic loosening [1]. The discovery of calcitonin gene-related peptide (CGRP) -immunoreactive nerve fibres in the interface membrane and elevated CGRP levels in synovial fluids of loosened arthoplasty suggested a linkage between the nervous system and aseptic loosening [2,3].

In our previous in-vivo study of alpha-CGRP deficient mice we demonstrated an influence of the neurotransmitter alpha-CGRP and the importance of the osteoprotegerin/receptor activator of nuclear factor-κB ligand/receptor activator of nuclear factorκB (OPG/RANKL/RANK) system on particle-induced osteolysis [4]. The neurotransmitter alpha-CGRP has multiple physiological roles. For example, it affects the metabolism of skeletal muscle, the liver and the kidneys, and inhibits glycogen synthesis [5,6]. It acts as a potent vasodilatator, as a neurotrophic effector and as a mediator in the neurogenic inflammatory response [7,8]. Alpha-CGRP receptors are expressed in brain tissue, adrenal and pituitary glands, the exocrine pancreas, peripheral tissue and on osteoblasts [9-11]. The OPG/RANKL/RANK system plays a key role in the cross-talk between osteoblasts and osteoclasts [12,13]. RANKL and OPG are members of a ligand-receptor system that directly regulates osteoclast differentiation and bone resorption, and both are produced and secreted by osteoblastic lineage cells [13,14]. On the one hand, RANKL binds to RANK, which is expressed on osteoclast progenitors, and leads to osteoclast activation. On the other hand, OPG binds to RANKL and thereby inhibits osteoclast activation. The osteoblast function can be described by alkaline phosphatase specific activity [15].

To closer study our in-vivo results, we analyzed MG-63 osteoblast like cells in the presence of wear particles and alpha-CGRP in-vitro.

## Methods

### Peptide

Alpha-CGRP (Sigma Aldrich, Cat. No. C0167, Saint Louis, Missouri, USA) was dissolved in 1% acetic acid or water and stored at -20°Celsius before use. During cell seeding, alpha-CGRP was added daily to the experimental wells to form different concentrations (10^-7 ^M, 10^-9 ^M or 10^-11 ^M), as introduced by Villa et al. while an alpha-CGRP-free medium was added to the control group [16].

### Preparation of wear particles

The commercially pure ultra-high molecular weight polyethylene (UHMWPE) particles (Ceridust VP 3610, Clariant, Gersthofen, Germany) with a mean particle size (given as equivalent circle diameter) of 1.74 ± 1.43 μm (range 0.05-11.06) were used in this study [17]. For endotoxin removal, the particles were treated for 24 hours with 99% ethanol at room temperature and were afterwards dried in a desiccator. The efficacy of the method was checked using Limulus Amebocyte Lysate (LAL) Assay (Charles River, Kent, United Kingdom) with a sensitivity of 0.25 EU/ml according to the manufacturer's directions. The test was found to be negative. Subsequently, particles were re-suspended in 10% endotoxin-free fetal calf serum (FCS), vortexed and treated in a sonicating water bath. Flow cytometry was used to measure the number of particles per unit volume of solution.

### MG-63 cells

The human osteoblast-like MG-63 cell line (CRL-1427™, ATCC) was obtained from the American Type Culture Collection. The cell line was cultured in RPMI 1640 medium (PAA, Pasching, Austria), supplemented with 100 U of penicillin G/ml (Gibco, BRL, Eggenstein, Germany), 100 μg of streptomycin/ml (Gibco), 2 mM L-glutamine (Gibco) and 10% fetal calf serum (PCS) at 37°C in a humidified atmosphere (5% CO2 and 95% air).

For the experiment, MG-63 cells were seeded into 6-well flat bottomed culture plates at the quantity of approximately 1.5 × 10^5 ^cells per well. After 24 hours, an 80% confluence of the cells was reached. The supernatant was removed and a fresh medium containing UHMWPE particles was added. During this procedure, different quantities of particles were added to form two different cell-particle ratios (1:100 and 1:500).

### Isolation of RNA and quantitative Real Time RT-PCR analysis

Total RNA was isolated using Qiashraddle (Qiagen, Hilden, Germany) and purified using the RNeasy Mini Kit (Qiagen, Hilden, Germany). Both procedures were performed according to the manufacturer's specification. The purification included a DNase treatment using the RNase free DNase Set (Qiagen, Hilden, Germany). The yield and purity of the RNA was measured photometrically. RNA was analyzed by quantitative real time polymerase chain reaction (RT-PCR) in a Rotorgene Cycler (Corbett Research, Mortlake, Australia) using the QuantiFast SYBR Green RT-PCR kit (Qiagen, Hilden, Germany) according to the manufacturer's instructions. A conventional PCR was performed to obtain a product of amplification suitable for the construction of standard curves with the real-time PCR procedures. The incorporation of Sybr Green into the PCR products was monitored in real time after each PCR cycle, resulting in the calculation of the threshold cycle or C_t _value that defines the PCR cycle number at which an exponential growth of PCR products begins. PCR cycle conditions were as follows: 10 minutes at 50°C, 5 minutes at 95°C, 35 to 40 cycles of 10 seconds at 95°C and 30 seconds at 60°C. Each PCR procedure included a negative control reaction without a template. To exclude residual DNA contamination of the RNA samples, RT-PCR was also performed without reverse transcriptase. For mRNA amplification, the validated primers were obtained from Qiagen (Qiagen, Hilden, Germany): β-actin (Cat. No. QT00095431), RANKL (Cat. No. QT00215614) and OPG (Cat. No. QT00014294). The PCR products were sequenced and found to be identical to the published sequences. The β-actin housekeeping gene was used as reference for the relative quantification of the gene of interest, which was expressed as the ratio of 'concentration of the target' to 'concentration of β-actin'.

### Western Blot

MG-63 cells were stimulated with and without particles (the cell-particle ratios were 1:100 and 1:500 respectively) for 24, 48, 72 hours. The cells were washed with ice-cold phosphate-buffered saline (PBS) twice and directly lysed in Laemmli buffer. The lysate was sonicated, boiled for 5 minutes and centrifuged at 16,000 g for 10 minutes at 4°C. The supernatant was recovered as total cell lysate, sub-packaged and stored at -80°C. Equal amounts of protein (10 μg) were separated by 8% SDS-PAGE and electro-transferred to 0.45 μm polyvinylidene difluoride membranes (Millipore, Bedford, USA). Following transfer, membranes were blocked with a solution of 0.1% Tween 20/TBS (TBS/T) containing 5% non-fat milk for one hour at room temperature and then incubated with monoclonal mouse anti-human OPG antibody (GTX11994, GeneTex, USA, final dilution 1:300) or rabbit polyclonal human RANKL (AB1862, Chemicon, Temecula, California, USA, final dilution 1:3500) overnight at 4°C. Specifically bound primary antibodies were detected with peroxidase-coupled secondary antibody and enhanced chemiluminescence (Cell Signaling Technologies, Beverly, MA). The bands were visualized by nitroblue tetrazolium/5-bromo-4-chloro-3-indolyl-phosphate. Glyceraldehyde-3-Phosphate Dehydrogenase (GAPDH) was used as house keeping gene.

For densitometric analyzes, blots were scanned and quantified using Quantity One analysis software (Bio-Rad, Hercules, CA, USA). The results were expressed as the percentage of GAPDH immunoreactivity.

### Alkaline phosphatase specific activity

Upon termination of culture, the medium was carefully aspirated from each well. The QuantiChromeTM Alkaline Phosphatase Assay Kit (Cat. No. DALP-250; BioAssay Systems, Hayward, CA) was used to measure alkaline phosphatase (AP) activity levels in lysate samples of 10^4 ^cells, following the manufacturer's instructions.

### Statistical analysis

Results from representative experiments are shown. They were expressed as mean ± standard deviation. A repeated measurement ANOVA for all continuous dependent variables determined if there was (a) a time-by-group interaction effect, (b) a time effect and (c) inter-group effect. When F-values corresponding to a time-by-group interaction effect for a given variable were found to be significant, simple effects testing was performed to determine a time effect within each experimental group. Subsequently, one-way ANOVA tests were used to determine the detectable change between the groups at each time point. One-way ANOVA tests, at each time point relative to the previous time point, determined if there were significant changes from each time-point. A p-value < 0.05 was considered to indicate statistical significance.

## Results

**Figure 1:**
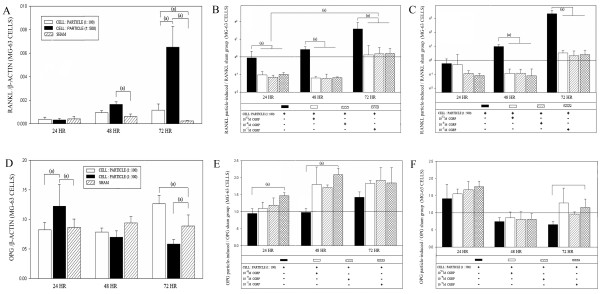
**RANKL and OPG mRNA levels in MG-63 cells after particle and alpha-CGRP treatment**. Time course of (A) RANKL and (D) OPG mRNA expression of MG-63 cells after stimulation with different UHMWPE particle concentrations. Time course of UHMWPE particle-induced RANKL mRNA expression after treatment with different alpha-CGRP doses in (B) a cell-particle ratio of 1:100 and (C) a cell-particle ratio of 1:500. Time course of UHMWPE particle-induced OPG mRNA expression after treatment with different alpha-CGRP doses in (E) a cell-particle ratio of 1:100 and (F) a cell-particle ration of 1:500. Significant differences are marked. ((a) P < 0.05).

RANKL mRNA expression of MG-63 cells was significantly elevated in high particle concentrations after 48 hours and in both particle concentrations after 72 hours (Figure 1A).

In the cell-particle ratio of 1:100 RANKL mRNA expression was significantly decreased by all concentrations of alpha-CGRP at all time-points (P < 0.05) (Figure 1B). A significant effect of time on RANKL mRNA expression inhibited by different concentrations of alpha-CGRP was found.

In particle concentrations of 1:500 RANKL mRNA expression was significantly decreased by all concentrations of alpha-CGRP after 48 and 72 hours (P < 0.05) (Figure 1C). The effect of time on RANKL mRNA expression in cell particle ratio of 1:500 inhibited by different concentrations of alpha-CGRP was not significant (p = 0.09). No significant differences of inhibition between the tested alpha-CGRP concentrations in both particle concentrations were revealed.

The time course of OPG mRNA expression in MG-63 cells differed after treatment with cell-particle concentrations of 1:100 and 1:500 (Figure 1D). In cell-particle concentrations of 1:100 OPG mRNA-expression significantly increased after 72 hours (P < 0.05). In cell-particle concentrations of 1:500 a significant increase of OPG mRNA was found after 24 hours turning to a significant decrease after 72 hours.

Alpha-CGRP stimulation in cell-particle concentrations of 1:100 lead to an up-regulation of OPG mRNA. These results were significant in high (10^-7 ^M) alpha-CGRP concentrations after 24 and 48 hours (P < 0.05) (Figure 1E). In cell particle concentrations of 1:500 a significant up-regulation of OPG mRNA was found after treatment with high (10^-7 ^M) alpha-CGRP concentrations after 72 hours (Figure 1F).

**Figure 2:**
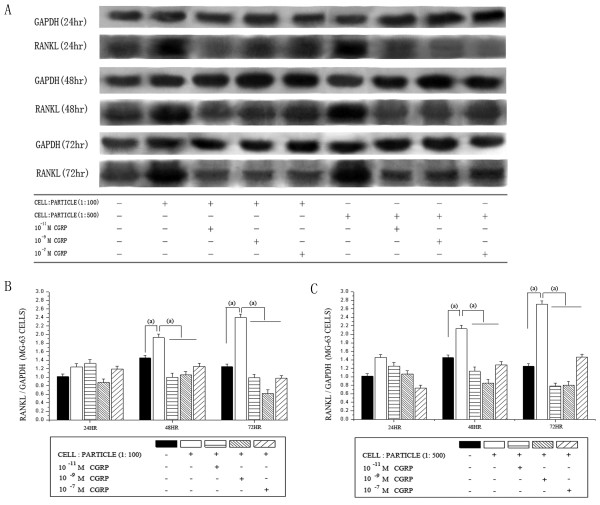
**RANKL protein levels in MG-63 cells after particle and alpha-CGRP treatment**. Time courses of RANKL protein levels in MG-63 osteoblast-like cells stimulated by UHMWPE particles and alpha-CGRP using Western blot analysis. (A) Representative Western blot for RANKL in untreated group and the alpha-CGRP-incubated groups. Densitometric quantification of RANKL in (B) a cell-particle ratio of 1:100 and (C) a cell-particle ratio of 1:500 with and without alpha-CGRP-incubation. RANKL protein levels are expressed relatively to GAPDH. Data are reported as mean ± standard deviation (n = 5). ((a) P < 0.05).

The detected RANKL protein levels detected by Western blot analysis showed a significant increase in both particle groups after 48 and 72 hours (Figure 2). Alpha-CGRP treatment lead to a significant decrease of RANKL protein after 48 and 72 hours. The analyzed alpha-CGRP concentrations did not show significantly differences.

**Figure 3:**
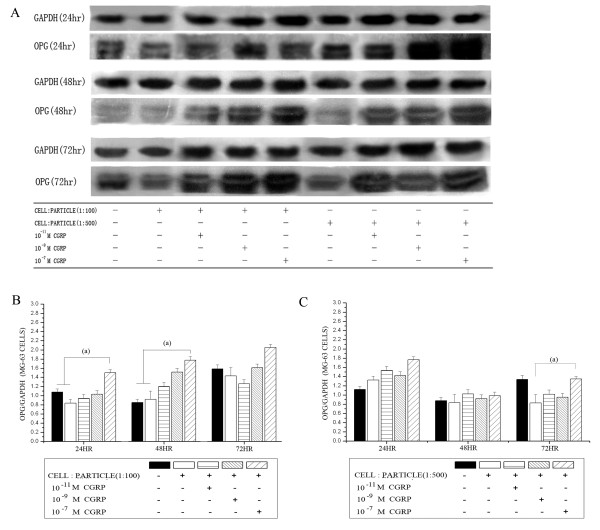
**OPG protein levels in MG-63 cells after particle and alpha-CGRP treatment**. Time courses of OPG protein levels in MG-63 osteoblast-like cells stimulated by UHMWPE particles and alpha-CGRP using Western blot analysis. (A) Representative Western blot for OPG in untreated group and the alpha-CGRP-incubated groups. Densitometric quantification of OPG in (B) a cell-particle ratio of 1:100 and (C) a cell-particle ratio of 1:500 with and without alpha-CGRP-incubation. OPG protein levels are expressed relatively to GAPDH. Data are reported as mean ± standard deviation (n = 5). ((a) P < 0.05).

Western blot analysis in a cell-particle ratio of 1:100 showed significantly elevated OPG protein levels after 24 and 48 hours in high alpha-CGRP concentrations. (Figure 3B). In the cell-particle ratio of 1:500 a significantly elevated OPG protein was found after 72 hours (Figure 3C). The significant changes of RANKL and OPG mRNA corresponded to the detected protein levels (compare Figure 1, 2, 3).

To further show a possible dose-dependent influence of alpha-CGRP we analyzed the OPG/RANKL mRNA ratio. In cell-particle concentrations of 1:100 after 24 hours, a significantly higher (P < 0.05) OPG/RANKL mRNA ratio was found after treatment with high (10^-7^M) alpha-CGRP concentrations compared to low (10^-11^M) alpha-CGRP concentrations. The OPG/RANKL mRNA ratio was not found to be different in cell/particle ratios of 1:500. After 48 and 72 hours the OPG/RANKL mRNA ratio was not different between the tested alpha-CGRP concentrations.

AP activity was significantly (P < 0.05) decreased in cell-particle ratios of 1:100 compared to the control group at all time points (Table 1). Cell-particle ratios of 1:500 lead to a significantly (P < 0.05) decreased AP activity compared to the 1:100 group and the control at all time points. All alpha-CGRP concentrations did not change AP activity significantly at the analyzed time points.

**Table 1:** Alkaline phosphatase specific activity of MG-63 cells incubated with alpha-CGRP

	1:100	1:100; 10^-11 ^M CGRP	1:100; 10^-9 ^M CGRP	1:100; 10^-7 ^M CGRP	1:500	1:500; 10^-11^M CGRP	1:500; 10^-9 ^M CGRP	1:500; 10^-7 ^M CGRP
24 h	0.84 ± 0.02	0.82 ± 0.01	0.82 ± 0.03	0.82 ± 0.03	0.75 ± 0.04	0.73 ± 0.02	0.72 ± 0.04	0.73 ± 0.03

48 h	0.82 ± 0.04	0.81 ± 0.04	0.82 ± 0.02	0.80 ± 0.04	0.71 ± 0.02	0.71 ± 0.05	0.71 ± 0.03	0.71 ± 0.04

72 h	0.81 ± 0.03	0.80 ± 0.02	0.79 ± 0.03	0.79 ± 0.04	0.69 ± 0.03	0.70 ± 0.04	0.70 ± 0.04	0.68 ± 0.03

Alkaline phosphatase specific activity of MG-63 cells incubated with different cell-particle ratios (1:100, 1:500) and different doses of alpha-CGRP (10^-7 ^M, 10^-9 ^M, 10^-11 ^M). Treatment/control ratios (T/C) are shown. Data are reported as mean ± standard deviation.

## Discussion

Until now the effects of alpha-CGRP in polyethylene particle-induced osteolysis have not been described in detail. This is the first time the effect of alpha-CGRP on RANKL and OPG mRNA expression has been examined in vitro under particle incubation. Our finding gives further support to previous reports of a linkage between neurotransmitters and particle-induced osteolysis [2,4,18].

This study shows a possible interaction of osteoblasts via the OPG/RANKL/RANK system after UHMWPE particle and alpha-CGRP. A dysbalanced interaction between osteoclasts and osteoblasts via the OPG/RANKL/RANK system might be one reason for dose-dependent particle-induced osteolysis. Our results show a significant RANKL up-regulation by particles and a significant dose-dependent down-regulation of RANKL production by alpha-CGRP. The OPG results did not show an inverse proportion of the RANKL levels as we believed. The detected OPG levels in the cell-particle ratio of 1:500 correspond to the literature whereas the elevation of OPG in the cell-particle ratio of 1:100 after 72 hours does not. The up-regulation of OPG was found after alpha-CGRP treatment in all doses at 24, 48 and 72 hours, but they did not reach significance in most groups. Analog to the literature, high RANKL levels and low OPG levels can cause a stimulation of osteoclasts [12,19]. The differing OPG results might be of small relevance for the in-vivo interaction as our results show a stronger influence of particles and alpha-CGRP on RANKL than on OPG. The results of our study correspond to the findings that macrophage-osteoclast differentiation occurs in the presence of soluble RANKL and that this process is inhibited by OPG [20]. We suggest that the discrepancy between RANKL and OPG mRNA expression of osteoblasts affected by UHMWPE particles is one of the reasons for periprosthetic osteolysis. The dual alpha-CGRP-influenced enhancers of bone formation showing down-regulated RANKL and an up-regulated OPG could theoretically inhibit the differentiation and activity of osteoclasts.

In the past decade, opinions regarding the influence of the neurotransmitter alpha-CGRP on bone metabolism have been controversial. On the one hand, alpha-CGRP has been shown to be a physiological activator of bone formation [21]. Cornish et al. found that osteoblasts respond to alpha-CGRP by increased growth [22]. Transgenic mice with an over-expression of alpha-CGRP present a phenotype of increased trabecular bone volume caused by an increased bone formation rate due to osteoblast activity [23]. Alpha-CGRP knock-out mice show a phenotype of decreased bone formation and osteopenia [24]. Moreover, alpha-CGRP inhibits the differentiation and recruitment of osteoclast precursors [25,26]. On the other hand, increased trabecular bone volume and reduced osteopenia were found in mice lacking both alpha-CGRP and calcitonin [27]. In this study, the effects of alpha-CGRP on the analyzed OPG/RANKL/RANK system might activate bone formation analog to the cell culture experiment of Cornish et al. and the transgenic mice of Ballica et al. and Schinke et al. [22-24,28]. We analyzed the AP specific activity to further show an established marker of osteoblast function as described by Dean et al. [15]. The decreasing AP activity shows the particle-dose dependent reduction of activity of MG-63 osteoblast-like cells. The non-significant AP activity reaction to different alpha-CGRP levels might be due to the analyzed time course. Chen et al. found the maximal AP activity after 25 days in MG-63 cells [29]. Further studies should focus on the time course of AP and further osteoblast specific markers to better understand the reaction of osteoblasts on alpha-CGRP.

A complex of other factors besides the analyzed OPG/RANK/RANKL system is involved in bone formation, activation and survival of osteoblasts and osteoclasts. Several pathways regulating the function of bone remodeling have been reported in the past, including TNF-alpha/TNFR/TRAF1 and IL-6/CD126/JAK/STAT [30,31]. Osteoblast activity is strongly regulated by surrounding pH and growth factors released from resorbed bone matrix that stimulate osteoblasts to promote or inhibit bone formation [32,33]. This may have an impact on the bone mass outcome at each remodeling cycle [34]. Furthermore, wear debris has a direct influence on macrophage-osteoclast differentiation. Human macrophages isolated directly from periprosthetic tissues surrounding loosened implants can differentiate into multinucleated cells and show all the functional and cytochemical characteristics of osteoclasts [35]. Therefore, it can be suggested that alpha-CGRP, by activating bone remodeling, may contribute to the precise adjustment of this process favoring the gain or loss of bone mass depending on the local environment. Finally, a discrepancy between bone formation and resorption due to alpha-CGRP might appear with the increase in the concentration of wear particles.

It remains uncertain whether our in vitro findings in osteoblast-like cells can be directly transferred to an up-regulation or down-regulation of bone formation in aseptic loosening of joint replacements. A limiting factor of this study is the partial focus on MG-63 osteoblast-like cells. These commercially available MG-63 osteosarcoma cells are often used as model for the osteoblastic phenotype because of their rapid growth and their homogeneity in the cell circle [36-40], but the OPG/RANKL/RANK system in vivo interacts between both osteoblasts and osteoclasts. The interaction of osteoclasts and osteoblasts might have a major impact on the later osteoprotective or catabolic result. Furthermore, this cell culture experiments has a limited perspective of time as we analyzed the MG-63 cells for only 72 hours. As aseptic loosening is a process which can take years, the reactions by osteoblasts might change in the course of time.

## Conclusion

In conclusion, the present study provides data describing the activation of signaling pathways in an osteoblast-like human cell under incubation with UHMWPE particles. Our data shows significant changes of RANKL, OPG, and AP activity due to UHMWPE particles and alpha CGRP supporting the concept of a linkage between the peripheral nervous system and aseptic loosening. Our results improve the understanding of alpha-CGRP having an osteoprotective influence on particle-induced osteolysis via the OPG/RANKL/RANK-system. Further studies of the interaction of osteoclasts, osteoblasts, neurotransmitters, and the OPG/RANKL/RANK system have to be undertaken to gain a better understanding of the multifactorial process of aseptic loosening and possible therapeutic options.

## List of Abbreviations used

CGRP: calcitonin gene-related peptide CGRP; OPG: osteoprotegerin; RANK: receptor activator of nuclear factor-κB; RANKL: receptor activator of nuclear factorκB ligand; UHMWPE: ultra-high molecular weight polyethylene

## Competing interests

The authors declare that they have no competing interests.

## Authors' contributions

XJ and HJ have made substantial contributions to acquisition of data and analysis and interpretation of data, have been involved in drafting the manuscript and revising it critically for important intellectual content, and have given final approval of the version to be published. KM and WC have made substantial contributions to conception and design, analysis and interpretation of data, have been involved in drafting the manuscript and revising it critically for important intellectual content, and have given final approval of the version to be published.

## References

[B1] Bauer TW Particles and periimplant bone resorption Clin Orthop Relat Res 2002 405 138 143 10.1097/00003086-200212000-00016 12461365

[B2] Ahmed M Bergstrom J Lundblad H Gillespie WJ Kreicbergs A Sensory nerves in the interface membrane of aseptic loose hip prostheses J Bone Joint Surg Br 1998 80 151 155 10.1302/0301-620X.80B1.8138 9460973

[B3] Qian Y Zeng BF Zhang XL Jiang Y High levels of substance P and CGRP in pseudosynovial fluid from patients with aseptic loosening of their hip prosthesis Acta Orthop 2008 79 342 345 10.1080/17453670710015238 18622837

[B4] Wedemeyer C Neuerburg C Pfeiffer A Heckelei A Bylski D von Knoch F Schinke T Hilken G Gosheger G von Knoch M Polyethylene particle-induced bone resorption in alpha-calcitonin gene-related peptide-deficient mice J Bone Miner Res 2007 22 1011 1019 10.1359/jbmr.070408 17419680

[B5] Beaumont K Pittner RA Moore CX Wolfe-Lopez D Prickett KS Young AA Rink TJ Regulation of muscle glycogen metabolism by CGRP and amylin: CGRP receptors not involved Br J Pharmacol 1995 115 713 715 8548167 10.1111/j.1476-5381.1995.tb14991.x PMC1908511

[B6] Leighton B Foot EA The role of the sensory peptide calcitonin-gene-related peptide(s) in skeletal muscle carbohydrate metabolism: effects of capsaicin and resiniferatoxin Biochem J 1995 307 Pt 3 707 712 7741700 10.1042/bj3070707 PMC1136708

[B7] Wimalawansa SJ Calcitonin gene-related peptide and its receptors: molecular genetics, physiology, pathophysiology, and therapeutic potentials Endocr Rev 1996 17 533 585 8897024 10.1210/edrv-17-5-533

[B8] Wimalawansa SJ Amylin, calcitonin gene-related peptide, calcitonin, and adrenomedullin: a peptide superfamily Crit Rev Neurobiol 1997 11 167 239 9209829 10.1615/critrevneurobiol.v11.i2-3.40

[B9] Bjurholm A Kreicbergs A Brodin E Schultzberg M Substance P- and CGRP-immunoreactive nerves in bone Peptides 1988 9 165 171 10.1016/0196-9781(88)90023-X 2452430

[B10] Yamamoto I Kitamura N Aoki J Shigeno C Hino M Asonuma K Torizuka K Fujii N Otaka A Yajima H Human calcitonin gene-related peptide possesses weak inhibitory potency of bone resorption in vitro Calcif Tissue Int 1986 38 339 341 10.1007/BF02555747 3089556

[B11] Brain SD Williams TJ Inflammatory oedema induced by synergism between calcitonin gene-related peptide (CGRP) and mediators of increased vascular permeability Br J Pharmacol 1985 86 855 860 2416378 10.1111/j.1476-5381.1985.tb11107.x PMC1916626

[B12] Khosla S Minireview: the OPG/RANKL/RANK system Endocrinology 2001 142 5050 5055 10.1210/en.142.12.5050 11713196

[B13] Yasuda H Shima N Nakagawa N Mochizuki SI Yano K Fujise N Sato Y Goto M Yamaguchi K Kuriyama M Identity of osteoclastogenesis inhibitory factor (OCIF) and osteoprotegerin (OPG): a mechanism by which OPG/OCIF inhibits osteoclastogenesis in vitro Endocrinology 1998 139 1329 1337 10.1210/en.139.3.1329 9492069

[B14] Hofbauer LC Khosla S Dunstan CR Lacey DL Boyle WJ Riggs BL The roles of osteoprotegerin and osteoprotegerin ligand in the paracrine regulation of bone resorption J Bone Miner Res 2000 15 2 12 10.1359/jbmr.2000.15.1.2 10646108

[B15] Dean DD Schwartz Z Liu Y Blanchard CR Agrawal CM Mabrey JD Sylvia VL Lohmann CH Boyan BD The effect of ultra-high molecular weight polyethylene wear debris on MG63 osteosarcoma cells in vitro J Bone Joint Surg Am 1999 81 452 461 10.1302/0301-620X.81B3.8758 10225790

[B16] Villa I Mrak E Rubinacci A Ravasi F Guidobono F CGRP inhibits osteoprotegerin production in human osteoblast-like cells via cAMP/PKA-dependent pathway Am J Physiol Cell Physiol 2006 291 C529 537 10.1152/ajpcell.00354.2005 16611736

[B17] von Knoch M Sprecher C Barden B Saxler G Loer F Wimmer M Size and shape of commercially available polyethylene particles for in-vitro and in-vivo-experiments Z Orthop Ihre Grenzgeb 2004 142 366 370 10.1055/s-2004-822589 15250012

[B18] Ren W Wu B Peng X Hua J Hao HN Wooley PH Implant wear induces inflammation, but not osteoclastic bone resorption, in RANK(-/-) mice J Orthop Res 2006 24 1575 1586 10.1002/jor.20190 16779834

[B19] Jacobs JJ Roebuck KA Archibeck M Hallab NJ Glant TT Osteolysis: basic science Clin Orthop Relat Res 2001 393 71 77 10.1097/00003086-200112000-00008 11764373

[B20] Itonaga I Sabokbar A Murray DW Athanasou NA Effect of osteoprotegerin and osteoprotegerin ligand on osteoclast formation by arthroplasty membrane derived macrophages Ann Rheum Dis 2000 59 26 31 10.1136/ard.59.1.26 10627423 PMC1752988

[B21] Naot D Cornish J The role of peptides and receptors of the calcitonin family in the regulation of bone metabolism Bone 2008 43 813 818 10.1016/j.bone.2008.07.003 18687416

[B22] Cornish J Callon KE Lin CQ Xiao CL Gamble GD Cooper GJ Reid IR Comparison of the effects of calcitonin gene-related peptide and amylin on osteoblasts J Bone Miner Res 1999 14 1302 1309 10.1359/jbmr.1999.14.8.1302 10457262

[B23] Ballica R Valentijn K Khachatryan A Guerder S Kapadia S Gundberg C Gilligan J Flavell RA Vignery A Targeted expression of calcitonin gene-related peptide to osteoblasts increases bone density in mice J Bone Miner Res 1999 14 1067 1074 10.1359/jbmr.1999.14.7.1067 10404006

[B24] Schinke T Liese S Priemel M Haberland M Schilling AF Catala-Lehnen P Blicharski D Rueger JM Gagel RF Emeson RB Amling M Decreased bone formation and osteopenia in mice lacking alpha-calcitonin gene-related peptide J Bone Miner Res 2004 19 2049 2056 10.1359/jbmr.040915 15537449

[B25] Akopian A Demulder A Ouriaghli F Corazza F Fondu P Bergmann P Effects of CGRP on human osteoclast-like cell formation: a possible connection with the bone loss in neurological disorders? Peptides 2000 21 559 564 10.1016/S0196-9781(00)00185-6 10822112

[B26] Zaidi M Fuller K Bevis PJ GainesDas RE Chambers TJ MacIntyre I Calcitonin gene-related peptide inhibits osteoclastic bone resorption: a comparative study Calcif Tissue Int 1987 40 149 154 10.1007/BF02555699 3105845

[B27] Huebner AK Schinke T Priemel M Schilling S Schilling AF Emeson RB Rueger JM Amling M Calcitonin deficiency in mice progressively results in high bone turnover J Bone Miner Res 2006 21 1924 1934 10.1359/jbmr.060820 17002587

[B28] Cornish J Callon KE Bava U Kamona SA Cooper GJ Reid IR Effects of calcitonin, amylin, and calcitonin gene-related peptide on osteoclast development Bone 2001 29 162 168 10.1016/S8756-3282(01)00494-X 11502478

[B29] Chen FP Hsu T Hu CH Wang WD Wang KC Teng LF Expression of estrogen receptors alfa and beta mRNA and alkaline phosphatase in the differentiation of osteoblasts from elderly postmenopausal women: comparison with osteoblasts from osteosarcoma cell lines Taiwan J Obstet Gynecol 2006 45 307 312 10.1016/S1028-4559(09)60248-5 17175487

[B30] Dempsey PW Doyle SE He JQ Cheng G The signaling adaptors and pathways activated by TNF superfamily Cytokine Growth Factor Rev 2003 14 193 209 10.1016/S1359-6101(03)00021-2 12787559

[B31] Rakshit DS Ly K Sengupta TK Nestor BJ Sculco TP Ivashkiv LB Purdue PE Wear debris inhibition of anti-osteoclastogenic signaling by interleukin-6 and interferon-gamma. Mechanistic insights and implications for periprosthetic osteolysis J Bone Joint Surg Am 2006 88 788 799 10.2106/JBJS.E.00711 16595469

[B32] Arnett TR Extracellular pH regulates bone cell function J Nutr 2008 138 415S 418S 18203913 10.1093/jn/138.2.415S

[B33] Sato S Futakuchi M Ogawa K Asamoto M Nakao K Asai K Shirai T Transforming growth factor beta derived from bone matrix promotes cell proliferation of prostate cancer and osteoclast activation-associated osteolysis in the bone microenvironment Cancer Sci 2008 99 316 323 10.1111/j.1349-7006.2007.00690.x 18271931 PMC11158371

[B34] Theoleyre S Wittrant Y Tat SK Fortun Y Redini F Heymann D The molecular triad OPG/RANK/RANKL: involvement in the orchestration of pathophysiological bone remodeling Cytokine Growth Factor Rev 2004 15 457 475 10.1016/j.cytogfr.2004.06.004 15561602

[B35] Mandelin J Liljestrom M Li TF Ainola M Hukkanen M Salo J Santavirta S Konttinen YT Pseudosynovial fluid from loosened total hip prosthesis induces osteoclast formation J Biomed Mater Res B Appl Biomater 2005 74 582 588 15768436 10.1002/jbm.b.30244

[B36] Langub MC Reinhardt TA Horst RL Malluche HH Koszewski NJ Characterization of vitamin D receptor immunoreactivity in human bone cells Bone 2000 27 383 387 10.1016/S8756-3282(00)00335-5 10962349

[B37] Parreno J Hart DA Molecular and mechano-biology of collagen gel contraction mediated by human MG-63 cells: involvement of specific intracellular signaling pathways and the cytoskeleton Biochem Cell Biol 2009 87 895 904 10.1139/O09-052 19935875

[B38] Wiontzek M Matziolis G Schuchmann S Gaber T Krocker D Duda G Burmester GR Perka C Buttgereit F Effects of dexamethasone and celecoxib on calcium homeostasis and expression of cyclooxygenase-2 mRNA in MG-63 human osteosarcoma cells Clin Exp Rheumatol 2006 24 366 372 16956425

[B39] Clover J Gowen M Are MG-63 and HOS TE85 human osteosarcoma cell lines representative models of the osteoblastic phenotype? Bone 1994 15 585 591 10.1016/8756-3282(94)90305-0 7873286

[B40] Luo XH Liao EY Su X Wu XP Parathyroid hormone inhibits the expression of membrane-type matrix metalloproteinase-1 (MT1-MMP) in osteoblast-like MG-63 cells J Bone Miner Metab 2004 22 19 25 10.1007/s00774-003-0442-6 14691682

